# Anilino-1,4-naphthoquinones as potent mushroom tyrosinase inhibitors: *in vitro* and *in silico* studies

**DOI:** 10.1080/14756366.2024.2357174

**Published:** 2024-05-30

**Authors:** Sahachai Sabuakham, Sutita Nasoontorn, Napat Kongtaworn, Thanyada Rungrotmongkol, Atit Silsirivanit, Ratchanok Pingaew, Panupong Mahalapbutr

**Affiliations:** a Department of Biochemistry, Center for Translational Medicine, Faculty of Medicine, Khon Kaen University, Khon Kaen, Thailand; b Program in Bioinformatics and Computational Biology, Graduate School, Chulalongkorn University, Bangkok, Thailand; c Center of Excellence in Structural and Computational Biology, Department of Biochemistry, Faculty of Science, Chulalongkorn University, Bangkok, Thailand; d Department of Chemistry, Faculty of Science, Srinakharinwirot University, Bangkok, Thailand

**Keywords:** Tyrosinase inhibition, anilino-14-naphthoquinones, molecular docking, molecular dynamics simulations

## Abstract

Tyrosinase, a pivotal enzyme in melanin synthesis, is a primary target for the development of depigmenting agents. In this work, *in vitro* and *in silico* techniques were employed to identify novel tyrosinase inhibitors from a set of 12 anilino-1,4-naphthoquinone derivatives. Results from the mushroom tyrosinase activity assay indicated that, among the 12 derivatives, three compounds (**1**, **5**, and **10**) demonstrated the most significant inhibitory activity against mushroom tyrosinase, surpassing the effectiveness of the kojic acid. Molecular docking revealed that all studied derivatives interacted with copper ions and amino acid residues at the enzyme active site. Molecular dynamics simulations provided insights into the stability of enzyme–inhibitor complexes, in which compounds **1**, **5**, and particularly **10** displayed greater stability, atomic contacts, and structural compactness than kojic acid. Drug likeness prediction further strengthens the potential of anilino-1,4-naphthoquinones as promising candidates for the development of novel tyrosinase inhibitors for the treatment of hyperpigmentation disorders.

## Graphical Abstract

**Figure:**
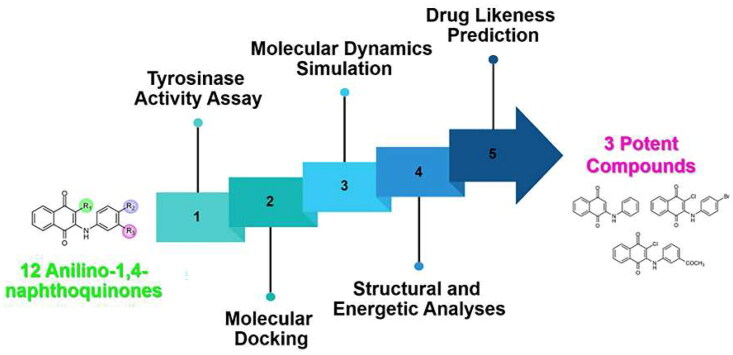

## Introduction

**Figure 1.**
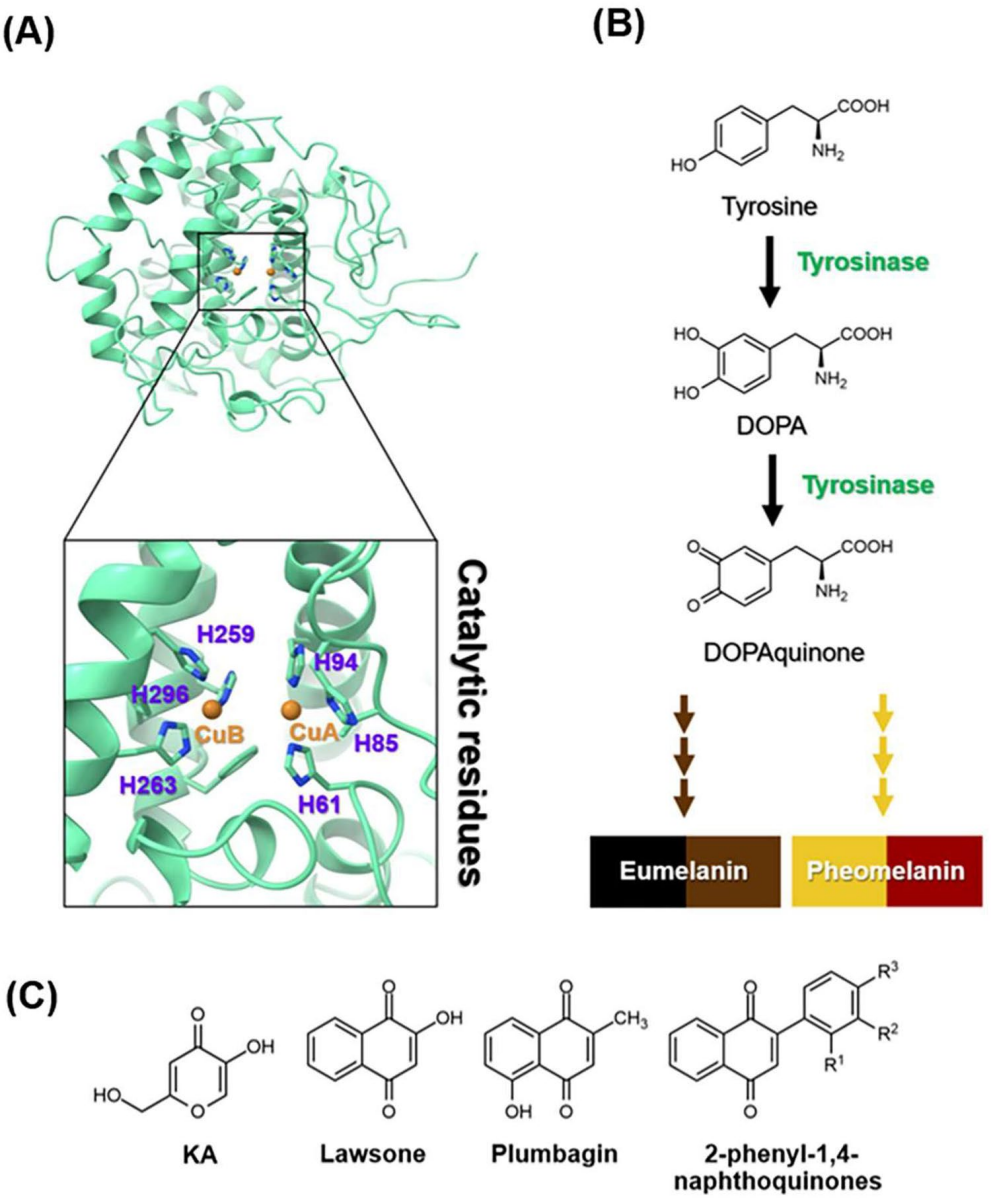
(A) Crystal structure of tyrosinase (PDB ID: 2Y9X^2^). The catalytic histidine residues and copper ions (CuA and CuB) were labelled in purple and orange, respectively. (B) Melanogenesis pathway produces eumelanin and pheomelanin^3^. (C) Chemical structures of KA, lawsone, plumbagin, and 2-phenyl-1,4-naphthoquinones.

The intricate process of melanin biosynthesis plays a predominant role in determining the colour of human skin, hair, and eyes^1^. Tyrosinase (Figure 1(A)) catalyses the rate-limiting step of melanin biosynthesis by converting tyrosine to DOPA and then to dopaquinone^4^, which serves as a precursor for the synthesis of eumelanin and pheomelanin^5^ (Figure 1(B)). Although melanin serves essential photoprotective functions, its dysregulation often results in undesirable hyperpigmentation disorders^6^, such as melasma and age spots. Consequently, the search for effective tyrosinase inhibitors has gained momentum, driven by both cosmetic and therapeutic interests^3^^,^^7^. Currently, there are a few known tyrosinase inhibitors, but most inhibitors have side effects and lack clinical efficacy^8^^,^^9^. Moreover, safety and stability are significant concerns when using tyrosinase inhibitors, particularly over extended periods and at high doses^10^. For instance, hydroquinone can undergo catabolism leading to benzene metabolites, posing potential toxicity to bone marrow, carcinogenicity^3^, skin irritation, and allergic reactions^11^. Arbutin has been reported to exhibit a counteractive effect on skin allergies^12^. The instability of kojic acid (KA, Figure 1(C)), especially during storage, has been observed^3^. Therefore, the search for novel potent tyrosinase inhibitors is needed.

Many lines of evidence have shown that 1,4-naphthoquinone derivatives (Figure 1(C)) can act as tyrosinase inhibitors. Lawsone can reduce melanin production by inhibiting tyrosinase activity and suppressing the expression of tyrosinase and microphthalmia-associated transcription factor^13^. Plumbagin was found to inhibit α-MSH-induced melanin synthesis in B16F10 melanoma cells by suppressing tyrosinase activity^14^. Additionally, 2-phenyl-1,4-naphthoquinones were reported to suppress both tyrosinase activity and melanin production in B16F10 murine melanoma cells^15^.

**Figure 2.**
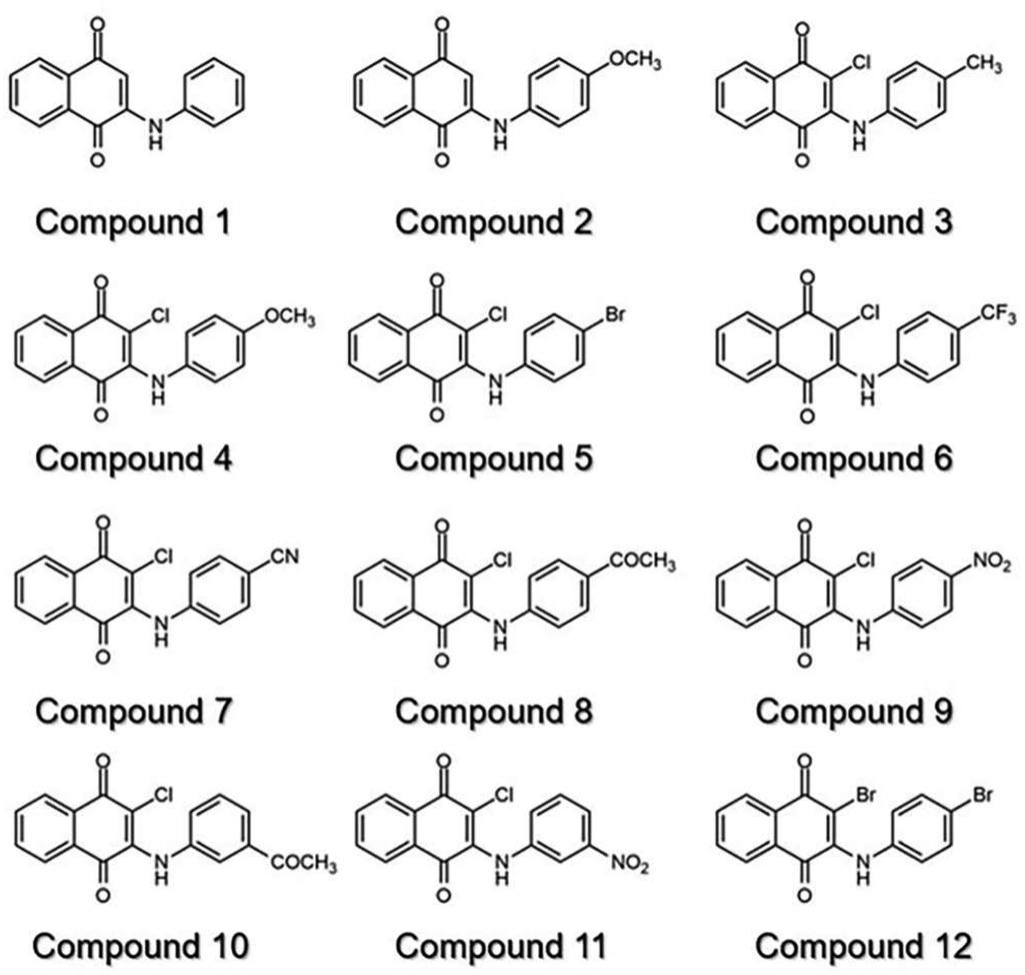
Chemical structure of 12 anilino-1,4-naphthoquinone derivatives.

Previously, we found that anilino-1,4-naphthoquinone derivatives (Figure 2), particularly compound **3**, possess anti-cancer potential by inhibiting EGFR activity with a fourfold higher potency than erlotinib^16^^,^^17^. However, the anti-tyrosinase activity of these derivatives has not been reported yet. Herein, a series of 12 anilino-1,4-naphthoquinone derivatives were chosen to explore their inhibitory capabilities against tyrosinase through both *in vitro* and *in silico* studies. Mushroom tyrosinase activity assay was initially utilised to identify potent compounds from 12 derivatives. Subsequently, molecular docking, molecular dynamics (MD) simulations, and free energy calculation based on the molecular mechanics/Poisson–Boltzmann surface area (MM/PBSA) method were conducted to elucidate the binding mechanism of the screened anilino-1,4-naphthoquinones against mushroom tyrosinase. The results obtained from this study could provide valuable insights for further design and development of novel tyrosinase inhibitors based on anilino-1,4-naphthoquinone scaffolds.

## Materials and methods

### Mushroom tyrosinase activity assay

Enzymatic conversion of l-DOPA into dopachrome by mushroom tyrosinase (Sigma Aldrich, St. Louis, MO) was detected using spectrophotometric evaluation. The mushroom tyrosinase activity assay was performed according to a previous report^18^. In this study, the enzyme assay was conducted at 37 °C, which is in the optimal temperature range of tyrosinase^19^, as per previous studies^20^^,^^21^. The enzyme, substrate, and inhibitor(s) were dissolved in potassium phosphate buffer at a pH of 6.8, which is in the optimal pH range of tyrosinase^22^. Note that, the solvent for preparing the stock solution of inhibitors was dimethyl sulphoxide. The 40 μL of mushroom tyrosinase (5 U/μL) was added to each well of a 96-well plate, followed by adding 50 μL of a prepared solution of 12 compounds and KA (Sigma Aldrich, St. Louis, MO) at various concentrations. Then, the substrate (10 μL of 50 mM of l-DOPA) was introduced to the samples, followed by incubation at 37 °C for 15 min in darkness. The absorbance was later measured at 450 nm. The experiment was conducted in triplicate. IC_50_ values were determined through dose–response curve analysis using GraphPad Prism 10.2.2 software (La Jolla, CA).

### Statistical analysis

Data are presented as the mean ± standard error of the mean (SEM) from three independent experiments (*n* = 3). Statistical comparisons between two groups were conducted using the *t*-test, with a *p* value of <0.05 considered to be statistically significant.

### System preparation and molecular docking

The crystal structure of tyrosinase from *Agaricus bisporus* (PDB ID: 2Y9X^2^) was retrieved from the Protein Data Bank. The protonation state of the protein was assessed at a pH of 7.4 using the PDB2PQR web server^23^. The covalent thioether bond between the carbon atom of H85 and the sulphur atom of C83 was constructed according to the previous report^2^. The 3D structures of 12 anilino-1,4-naphthoquinones and KA were constructed using the GaussView 6.1 program^24^. The protonation state of all investigated ligands was assessed at a pH of 7.4 using the Marvinsketch program software^25^. According to standard protocols^16^^,^^26–28^, the electrostatic potential (ESP) charges of each ligand were calculated with the HF/6-31G(d) level of theory using the Gaussian 09 program^29^. Molecular docking was conducted using the CB-DOCK2 server^30^. The 2D interaction profile was generated using the Discovery Studio visualiser. The docked complexes with the lowest docking energy were selected for MD simulations and free energy calculations.

### Molecular dynamics (MD) simulation

The all-atom MD simulation with a time step of 2 fs of each docked complex was performed using the AMBER16 software package^31^. The parameters for both bonded and non-bonded interactions of all inhibitors were handled using the general Amber force field (GAFF)^32^. The protein parameters were defined using the AMBER ff14SB force field^33^. The missing hydrogen atoms were added using the LEaP module. Each system was solvated using TIP3P water molecules^34^. To maintain neutrality, Na^+^ counterions were included. In the isobaric–isothermal (NPT) ensemble, a boundary condition was set with a constant pressure of 1 atm and a temperature of 310 K. The SHAKE algorithm^35^ was employed to constrain all bonds involving hydrogen. Non-bonded interactions were computed with a residue-based cut-off of 12 Å. The particle mesh Ewald method^36^ was utilised for handling long-range electrostatic interactions. To eliminate unfavourable interactions, we conducted 1000 iterations of the steepest descent (SD) method and 2000 iterations of conjugated gradient (CG) energy minimisation on the complex structure. Subsequently, the entire system was gradually heated to 310 K over a 100 ps. The systems underwent restrained MD simulations for a total of 5.0 ns, with progressively decreasing restraints of 50, 30, 20, 10, 5, and 1 kcal/mol·Å^2^. Afterward, the unrestrained MD simulations were conducted for 500 ps. Finally, MD simulations in the NPT ensemble (1 atm and 310 K) without any restraints were performed until reaching 10 ns.

### Structural and energetic analyses

The CPPTRAJ module^37^ of AMBER16 was used to compute structural parameters, including root mean square displacement (RMSD), the number of atom contacts (#Contacts), radius of gyration (Rg), and solvent accessible surface area (SASA) from the last 5 ns simulation. The obtained structural and energetic data were plotted using the OriginPro 2023 program. Additionally, hotspot residues involved in the binding process of ligand–protein complexes were determined using decomposition free energy calculation (Δ*G*_bind, res_) based on the MM/PBSA method^38^^,^^39^ with an interior dielectric constant of 5.0.

### Drug likeness prediction

Drug-likeness of all studied compounds was predicted using the SwissADME web tool (www.swissadme.ch)^40^.

## Results and discussion

### Anti-tyrosinase activity of anilino-1,4-naphthoquinones

**Figure 3.**
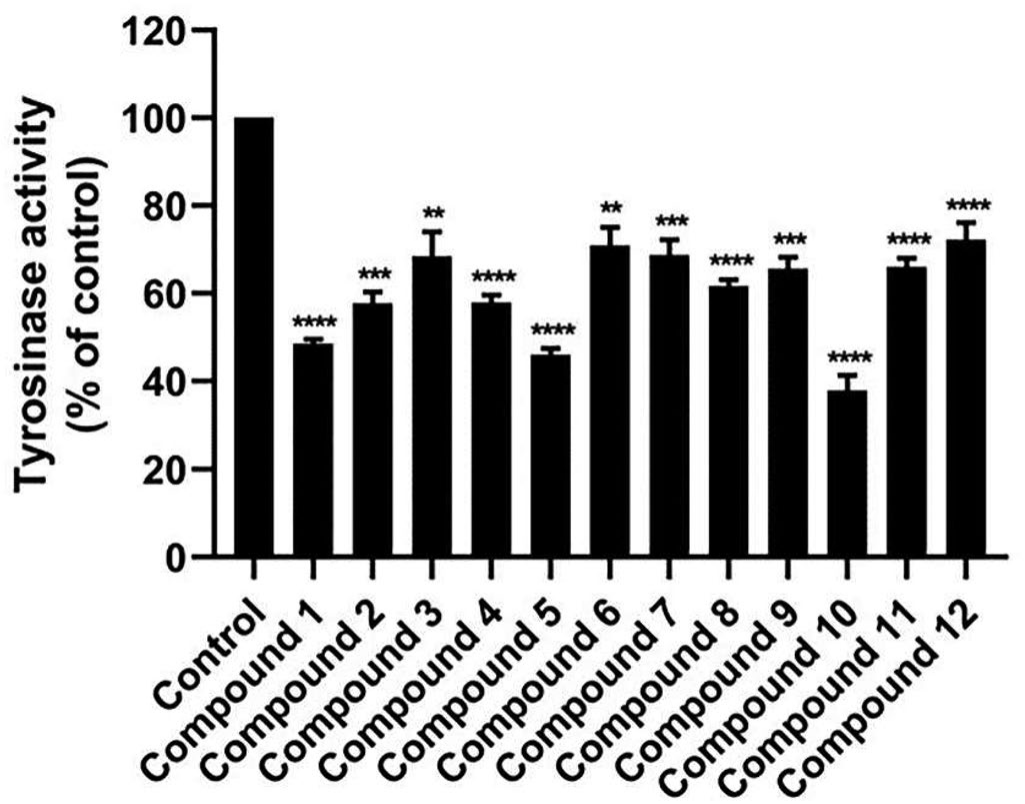
Mushroom tyrosinase inhibitory activity of 12 anilino-1,4-naphthoquinone derivatives at a concentration of 160 μM. Data are shown as the mean ± standard error of the mean (SEM) (*n* = 3). **p < 0.01, ***p < 0.001, and ****p < 0.0001 vs. control.

**Figure 4.**
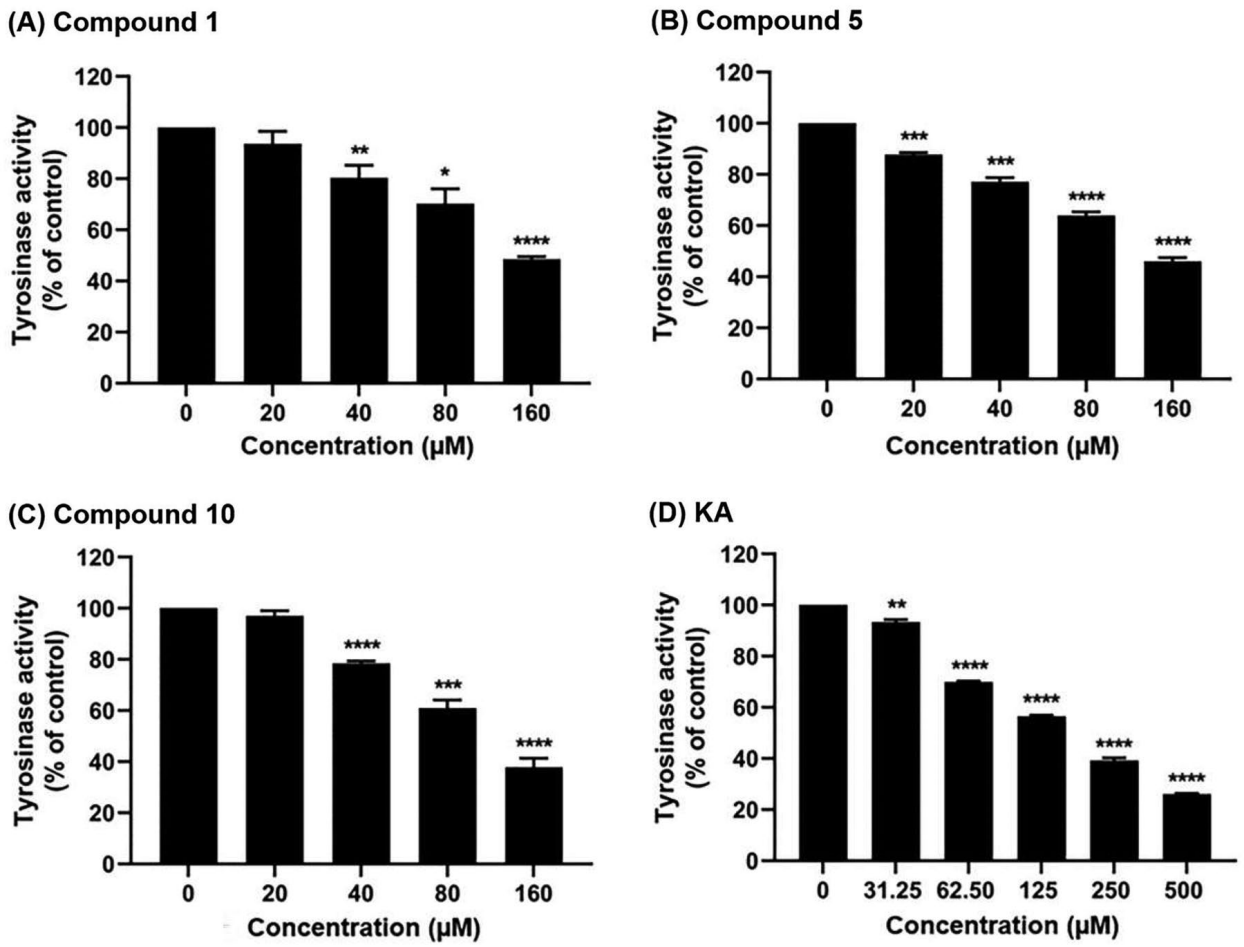
Tyrosinase inhibitory activity of (A) compound **1**, (B) compound **5**, (C) compound **10**, and (D) KA using l-DOPA as a substrate. Data are shown as the mean ± SEM (*n* = 3). *p < 0.05, **p < 0.01, ***p < 0.001, and ****p < 0.0001 vs. control.

The initial screening of the 12 synthesised anilino-1,4-naphthoquinone derivatives was conducted against tyrosinase at a concentration of 160 μM to identify compounds with potent inhibitory activity. Figure 3 illustrates that compounds **1** (49.58 ± 1.39%), **5** (46.05 ± 2.02%), and **10** (37.87 ± 4.96%) demonstrated higher anti-tyrosinase activity compared to the remaining derivatives (>50%). Therefore, these three potent compounds were selected to determine their half-maximal inhibitory concentration (IC_50_) value. As shown in Figure 4 and Table 1, all screened compounds inhibited tyrosinase activity in a concentration-dependent manner, similar to KA. Compound **1**, lacking functional groups on both the naphthoquinone and aniline moieties, inhibited tyrosinase activity with an IC_50_ value of 151.13 ± 4.39 μM. Introduction of bromine (–Br) on the aniline moiety and chlorine (–Cl) on the naphthoquinone ring (compound **5**) led to enhanced anti-tyrosinase activity (IC_50_ = 138 ± 6.07 μM). Notably, compound **10**, containing a –Cl substituent on the quinone ring and an acetyl group (–COCH_3_) on the aniline moiety, exhibited the highest tyrosinase inhibitory activity (IC_50_ = 111.95 ± 9.18 μM). The IC_50_ of KA was 168.90 ± 3.30 μM, which is similar to the previously reported IC_50_ values (235–250 μM^41^^,^^42^).

**Table 1.** IC_50_ values of compounds **1**, **5**, **10**, and KA against tyrosinase.

Compound	IC_50_ (μM)^a^
**1**	151.13 ± 4.39
**5**	138.00 ± 6.07
**10**	111.95 ± 9.18
KA	168.90 ± 3.30

^a^ Data are shown as the mean ± SEM of three independent experiments (*n* = 3).

It is worth noting that these three anilino-1,4-naphthoquinones showed greater inhibition than KA, as well as than the previously reported tyrosinase inhibitors, including morin (IC_50_ = 2320 μM)^43^, amphotericin B (IC_50_ = 263.36 ± 11.76 μM)^44^, glabrene (IC_50_ = 7600 μM)^45^, and methimazole derivatives (IC_50_ = 4100 μM) ^46^, indicating the potential for further development of anilino-1,4-naphthoquinones as novel tyrosinase inhibitors.

The highest anti-tyrosinase inhibitory effect of compound **10** was in good agreement with high structural stability and the compactness of the ligand–protein complex, as discussed later. Altogether, the three potent compounds (**1**, **5**, and **10**) were subjected to further analyses, including (i) molecular docking, (ii) MD simulation, (iii) key binding residues analysis, and (iv) drug likeness prediction.

**Figure 6.**
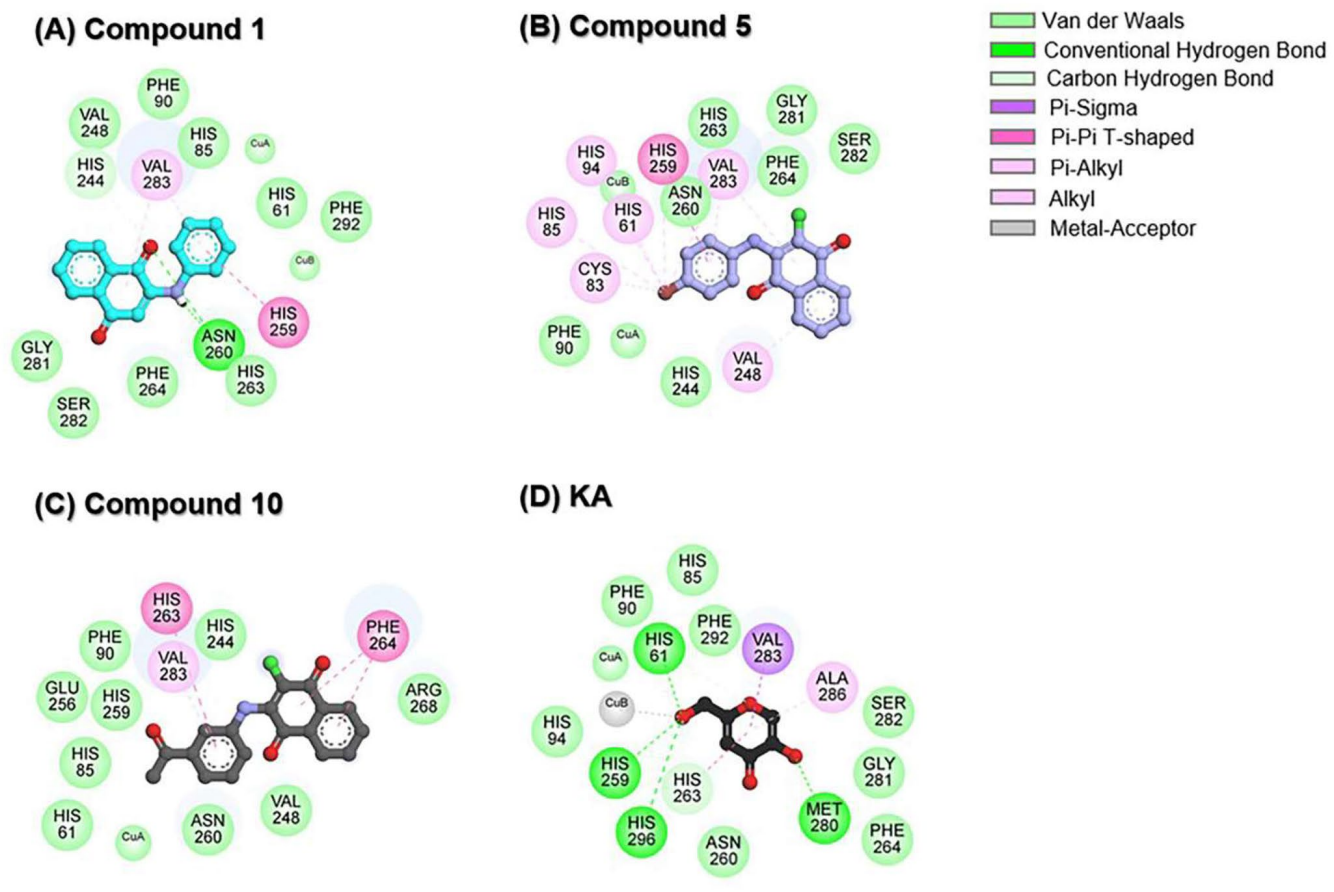
Time evolution of (A) RMSD and (B) Rg of compounds **1**, **5**, **10**, and KA in complexes with tyrosinase.

### Molecular docking

The binding mode between the screened compounds (**1**, **5**, and **10**) and KA in complexes with tyrosinase was studied using the CB-DOCK2 server. As shown in Table 2, the interaction energy of compounds **1** (−6.8 kcal/mol), **10** (−6.6 kcal/mol), and **5** (−6.1 kcal/mol) was lower than that of the KA–tyrosinase system (−5.3 kcal/mol).

**Table 2.** CB-DOCK2 interaction energy of compounds **1**, **5**, **10**, and KA against tyrosinase.

Compound	CB-DOCK2 interaction energy (kcal/mol)
**1**	−6.8
**5**	−6.1
**10**	−6.6
KA	−5.3

**Figure 5.**
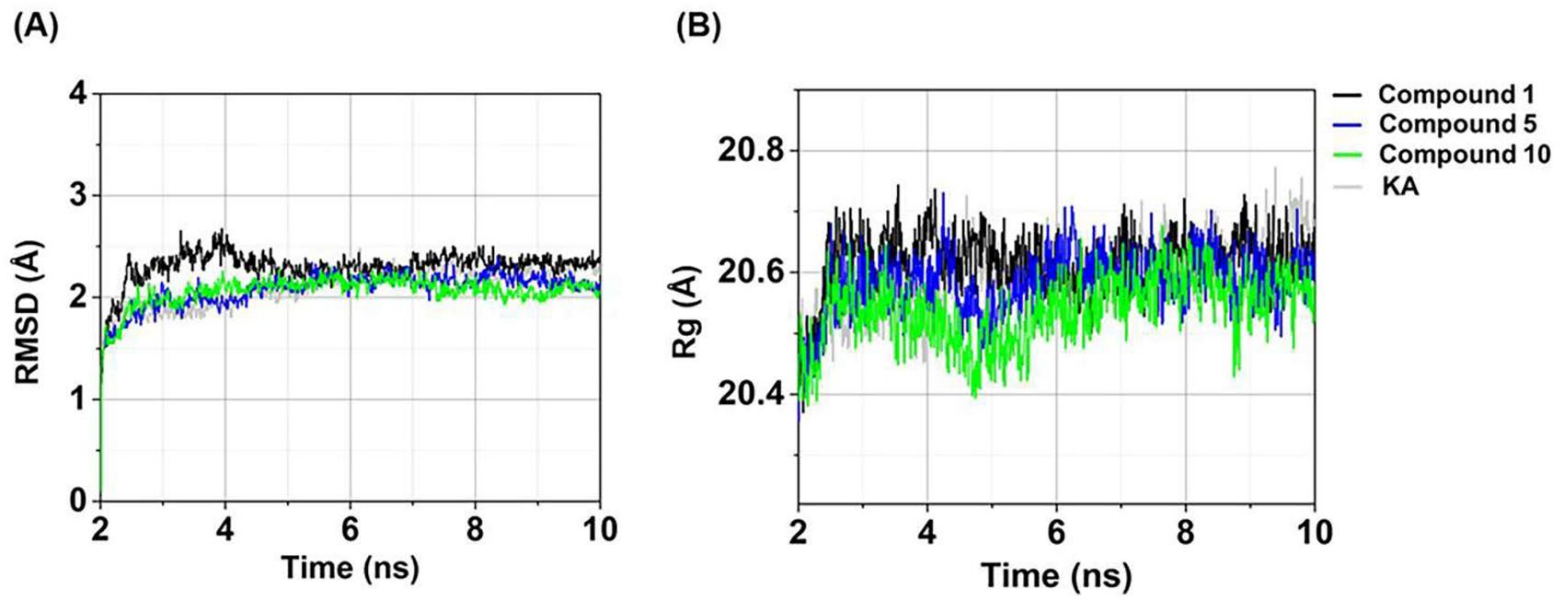
2D interaction profile of (A) compound **1**, (B) compound **5**, (C) compound **10**, and (D) KA in complexes with tyrosinase.

To delve into further detail, the two-dimensional (2D) interaction profile of each ligand within the active site of tyrosinase was analysed, and the results are depicted in Figure 5. Results revealed that three anilino-1,4-naphthoquinone derivatives could interact with the catalytic copper centre of tyrosinase. The −NH and C═O groups of compound **1** can form hydrogen bonds with N260 (Figure 5(A)), in good agreement with the previous study showing that Thiamidol™ had the capability to form hydrogen bond with N260 residue^47^. As compared to compound **1**, the substituted –Br atom of compound **5** can form halogen interactions with H61, C83, H85, H94, H259, and V283 residues (Figure 5(B)). Previous study by Paudel et al. indicated that an increase in the number of –Br moieties in the chemical structure correlates with the enhanced halogen interactions, contributing to their potent anti-tyrosinase activity^48^. For compound **10**, its –COCH_3_ substitution resulted in increased van der Waals (vdW) forces with catalytic residues H85 and H259 (Figure 5(C)). These structural findings support the increased tyrosinase inhibitory activity of compounds **5** and **10** when compared to compound **1** (Table 1).

The aromatic ring of three anilino-1,4-naphthoquinones engaged in Pi interactions with V248, H259, H263, F264, and V283 residues located in the active site of tyrosinase. The vdW interactions were found to mainly stabilise the binding between ligands and coppers/tyrosinase at the catalytic site, which was similar to previously reported luteolin and luteolin 5-O-β-d-glucopyranoside in complexes with tyrosinase^49^. In the case of KA–tyrosinase system, hydrogen bonds were found between the hydroxyl (−OH) groups of KA and the H61, H259, H296, and M280 residues (Figure 5(D)). The –OH group attached to the methylene (–CH_2_) group in KA was positioned close to the copper atoms, resembling the molecular docking simulation observed in previous reports between KA and tyrosinase^49^^,^^50^. It should be noted that the H61, H85, F90, H259, N260, H263, F264, and V283 residues found in KA–tyrosinase system were matched with those found in anilino-1,4-naphthoquinone–tyrosinase complexes. The binding of three potent anilino-1,4-naphthoquinones to the active site of tyrosinase is similar to the binding of 2-phenyl-1,4-naphthoquinones^15^, epicatechin^51^, catechin^52^, carvacrols^53^, benzimidazothiazolone analogs^54^, 2,3-dihydro-1,5-benzothiazepine derivatives^55^, kuwanon G, mulberrofuran G, and albanol B^56^ to the tyrosinase.

Although compounds **1**, **5**, and **10** lack the –OH functional group, their chemical structure contains other moieties (i.e. –Br, –COCH_3_, −NH, and C═O) that can form potential interactions with the tyrosinase as mentioned above. Supportively, previous reports have shown that several compounds lacking the –OH group (e.g. aryl pyrazoles, thiosemicarbazones, 5-benzylidene(thio)barbiturate-β-d-glycosides, and 3-/4-aminoacetophenones) exhibited higher tyrosinase inhibition than the well-known tyrosinase inhibitors, kojic acid and arbutin^57–62^.

Taken together, compounds **1**, **5**, and **10** can bind to the catalytic histidine residues (H61, H85, H94, H259, H263, and H296) and amino acid residues located in the active site of the tyrosinase. Additionally, they can interact with Cu ions (CuA and CuB). This finding suggested that our studied compounds could function as competitive inhibitors.

### Structural stability and compactness

The RMSD aids in the assessment of whether the simulated systems attain a state of stability. As shown in Figure 6(A), the RMSD values of the three anilino-1,4-naphthoquinones–tyrosinase complexes exhibited a gradual increase until reaching a stabilised level of approximately 2.11–2.32 Å after 5 ns. Similarly, the RMSD value for the KA–tyrosinase complex ultimately achieved a state of equilibrium following 5 ns, registering at 2.23 ± 0.09 Å. These outcomes implied that all studied systems achieved a state of equilibrium at 5 ns and maintained this stability until the conclusion of the MD simulations. Notably, the compound **10**–tyrosinase complex displayed the highest stability within the last 5 ns timeframe (RMSD of ∼2.11 ± 0.07 Å) compared to the other systems (RMSD of ∼2.17–2.32 Å).

We further computed the Rg values for all investigated systems to evaluate the compactness of the complex^63^. As illustrated in Figure 5(B), the Rg values for all systems were stable within a range of 20.46–20.62 Å after the last 5 ns, similar to the RMSD results (Figure 5(A)). Remarkably, compound **10** in complex with tyrosinase exhibited the highest compactness (Rg of ∼20.46 ± 0.04 Å within the last 5 ns) compared to the other systems (Rg of ∼20.54–20.62 Å).

Taken together, these three anilino-1,4-naphthoquinone–tyrosinase complexes, particularly compound **10**–tyrosinase complex, exhibited greater stability and compactness than the KA–tyrosinase system, which was in good agreement with the results of tyrosinase activity (Table 1).

### Atomic contacts and water accessibility

**Figure 7.**
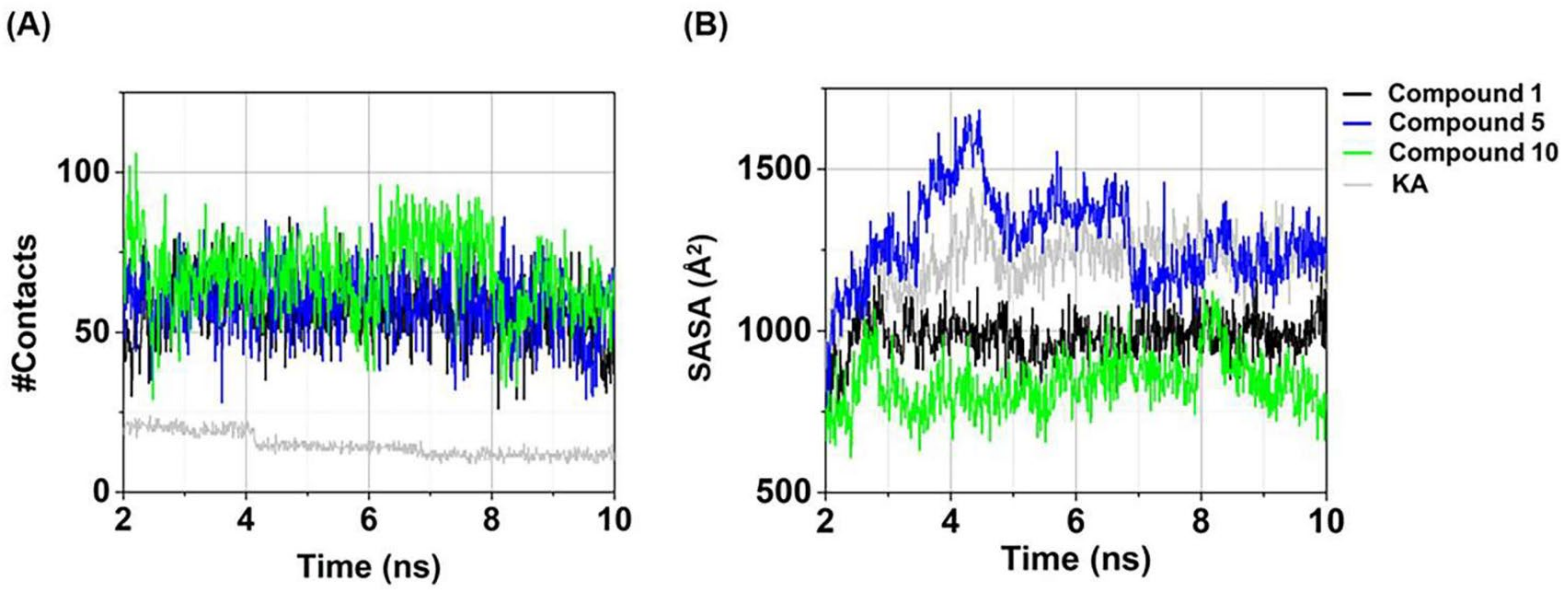
Time evolution of (A) #Contacts and (B) SASA of compounds **1**, **5**, **10**, and KA in complexes with tyrosinase.

Atomic contacts play a role in the protein–ligand binding affinity^64^. In this work, the #Contacts within a 4 Å radius of ligand was calculated for all systems. As shown in Figure 7(A), three anilino-1,4-naphthoquinones in complexes with tyrosinase exhibited the higher #Contacts (55.63 ± 8.67 to 67.76 ± 12.32, calculated from the last 5 ns) than the KA–tyrosinase complex (12.43 ± 1.63). Notably, compound **10**–tyrosinase system demonstrated the highest #Contacts (67.76 ± 12.32). These findings are strongly supported by the structural stability (Figure 6(A)) and the compactness in protein–ligand complex (Figure 6(B)) results as mentioned above.

SASA yields valuable insights into the exposed surface area of a ligand–protein complex, with a particular focus on elucidating the interactions with surrounding water molecules^64^^,^^65^. In this work, the amino acid residues of tyrosinase surrounding the ligand within a radius of 7.5 Å were taken into account, and the obtained results are presented in Figure 7(B). Among three anilino-1,4-naphthoquinones in complexes with tyrosinase, compound **10**–tyrosinase complex presented the lowest SASA (858.30 ± 79.40 Å^2^, calculated from the last 5 ns) compared to compound **1** (981.35 ± 58.51 Å^2^), compound **5** (1269.22 ± 98.84 Å^2^), and KA (1242.78 ± 61.11 Å^2^). These results were consistent with the aforementioned RMSD, Rg, #Contacts, and particularly with the experimental data on tyrosinase inhibitory activity.

### Key binding residues

**Figure 8.**
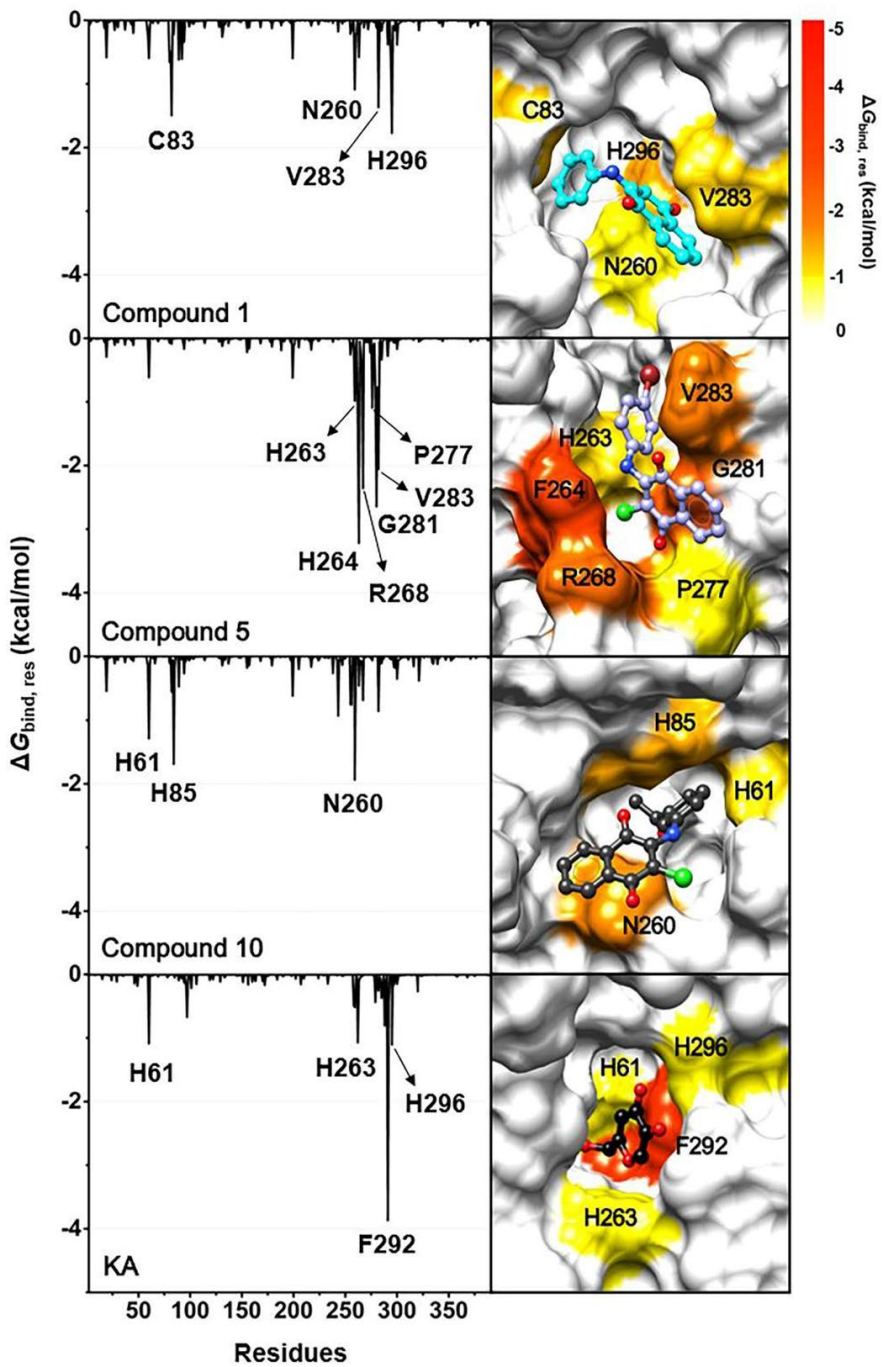
(Left) Δ*G*_bind, res_ of compounds **1**, **5**, **10**, and KA in complexes with tyrosinase. (Right) Representative structures showing the ligand orientation in the catalytic site drawn from the last 5 ns MD snapshots. The copper ions were hidden. The residues involved in the ligand binding (energy stabilisation of ≤ −1.0 kcal/mol) were coloured according to their Δ*G*_bind, res_ values, where the highest to lowest Δ*G*_bind, res_ values were shaded from yellow to red, respectively.

To explore the pivotal amino acid residues involved in the binding of three anilino-1,4-naphthoquinones to tyrosinase, the Δ*G*_bind, res_ calculation based on the MM/PBSA method^38^^,^^39^ was performed. This analysis was carried out using a selection of 100 snapshots derived from the last 5 ns of MD simulations, and the obtained results are shown in Figure 8. The Δ*G*_bind, res_ values of ≤ −1.0 kcal/mol were highlighted in this study.

Results suggested that amino acid residues that made substantial contributions to KA’s binding were H61, H263, F292, and H296, which align well with the binding patterns identified in a previous study on the interaction between KA and tyrosinase^50^. The compound **5**–tyrosinase complex exhibited the highest number of stabilising residues, including H263, F264, R268, P277, G281, and V283. The key binding residues for compound **1** and compound **10** were (i) C83, N260, V283, and H296 and (ii) H61, H85, and N260, respectively. To note that the H61, H263, and H296 residues found in KA–tyrosinase system were matched with those found in anilino-1,4-naphthoquinone–tyrosinase complexes. These aforementioned hotspot residues were also detected in the binding of 2-phenyl-1,4-naphthoquinones^15^, carvacrol derivatives^53^, benzimidazothiazolone derivatives^54^, 2,3-dihydro-1,5-benzothiazepine derivatives^55^, kuwanon G, mulberrofuran G, and albanol B^56^ to the active site of the tyrosinase. It should be noted that, all studied anilino-1,4-naphthoquinones can interact with the catalytic histidine residues in the enzyme active site, especially compound **10**, which could bind to the two catalytic H61 and H85 residues.

### Drug likeness prediction

Predicting the drug-likeness of compounds is a crucial element in drug development^66^. In this study, we employed the SwissADME web tool^40^ to predict the drug-likeness of three selected anilino-1,4-naphthoquinones together with KA. The parameters under investigation included molecular weight (MW), the number of hydrogen bond donors and acceptors (HBD and HBA), the number of rotatable bonds (RB), topological polar surface area (TPSA), and lipophilicity (log *P*). The results revealed that three compounds conformed to Lipinski’s rule of five criteria: (i) MW ≤500 Da, (ii) HBD ≤5 and HBA ≤10, (iii) RB ≤10, (iv) TPSA ≤140 Å^2^, and (v) log *P* ≤5^67^^,^^68^ as shown in Table 3. These findings suggested that our three compounds possess drug-likeness properties, laying the foundation for their potential advancement as novel drugs targeting the tyrosinase enzyme.

**Table 3.** Predicted values of drug-likeness parameters according to Lipinski’s rule of five criteria for compounds **1**, **5**, **10**, and KA.

Compound	Lipinski’s rule of five						
	MW (≤500 Da)	HBD (≤5)	HBA (≤10)	RB (≤10)	TPSA (≤140 Å^2^)	Log *P* (≤5)	Drug-likeness
**1**	249.26	1	2	2	46.17	1.68	Yes
**5**	326.61	1	2	2	46.17	2.55	Yes
**10**	325.75	1	3	3	63.24	1.47	Yes
KA	142.11	2	4	1	70.67	−1.69	Yes

MW: molecular weight; HBD: number of hydrogen bond donors; HBA: number of hydrogen bond acceptors; RB: number of rotatable bonds; TPSA: topological polar surface area; log *P*: lipophilicity.

## Conclusions

This study employed *in vitro* and *in silico* approaches to evaluate the inhibitory potential of 12 synthesised anilino-1,4-naphthoquinone derivatives against mushroom tyrosinase. Compounds **1**, **5**, and **10** exhibited superior anti-tyrosinase activity, surpassing the inhibitory activity of KA. Notably, compound **10** exhibited the highest anti-tyrosinase inhibitory activity, which was supported by the structural stability, the compactness of the ligand–protein complex, atomic contacts, and the water accessibility results. H61, C83, H85, N260, H263, F264, R268, P277, G281, V283, F292, and H296 were found to be hotspot residues for the binding of compounds **1**, **5**, **10**, and KA. All the compounds studied can interact with the catalytic histidine residues. The drug likeness prediction further strengthens the potential of compounds **1**, **5**, and especially **10** as promising candidates for the development of novel tyrosinase inhibitors. These findings pave the way for further development and potential application of anilino-1,4-naphthoquinones in dermatological and cosmetic products.

## Data Availability

The datasets presented in the current study are available upon reasonable request.
